# Co-creation of community-based innovations to improve access to malaria treatment in conflict-affected regions of Cameroon

**DOI:** 10.1186/s12936-026-05820-4

**Published:** 2026-03-12

**Authors:** Lundi-Anne Omam, Elvis Asangbeng Tanue, Esther Njomo Omam, Alain Metuge, Isabelle Nganmou, Coril Epie, Gilbert Kebam, Solomon Ako, Margaret Ebob Bessem, Helen Hawkings, Kolawole Maxwell, Yakouba Zoungrana, Elizabeth Berryman, Elizabeth Jarman, Bibiche Modjenpa Noukeme

**Affiliations:** 1 Center for Public Health Belfast, Queen’s University Belfast, Belfast, UK; 2 Department of Psychiatry, Cambridge Public Health Interdisciplinary Research Centre, University of Cambridge, Cambridge, Cambridgeshire UK; 3 Reach Out Cameroon (ROC), P.O Box 88, Buea, Cameroon; 4 Department of Public Health and Hygiene, Faculty of Health Sciences, University of Buea, P.O Box 63, Buea, Cameroon; 5 Konmofamba Actions Sans Frontieres (KASAFRO), Penja, Cameroon; 6 Malaria Consortium, The Green House, 244-254 Cambridge Heath Rd, London, E2 9UK UK; 7 Malaria Consortium Nigeria, No 33 Pope John Paul Street, Off Gana Street, Maitama, Abuja, FCT Nigeria; 8 Médecins Sans Frontières (MSF), Nairobi, Kenya

## Abstract

**Background:**

In conflict-affected regions of Cameroon, access to malaria care is severely hindered by displacement, insecurity, and disrupted health systems. In response, we conducted an operational research aimed at breaking barriers to malaria services in conflict-affected communities of Cameroon.

**Methods:**

In 2021, a participatory co-creation workshop was held on the 21st and 22nd of October 2021, bringing together stakeholders from government health services, community leaders, internally displaced persons (IDPs), and community health workers (CHWs)to collaboratively design interventions aimed at addressing barriers to accessing malaria treatment. The workshop built on prior formative research conducted in 80 conflict-affected communities across the South West and Littoral regions of Cameroon, which identified context-specific challenges to malaria care. The design process included plenary sessions, group discussions, and facilitated brainstorming, and employed participatory methods to ensure that community voices shaped the development of the interventions.

**Lessons learned:**

Three community-based innovations were co-created through this process. Community Health Participatory Approach (CoHPA) was designed to replace the traditional top-down community dialogue structure with a participatory, inclusive model. The Health Voucher System was designed to address financial and geographical barriers, a voucher-based system was introduced to enable access to subsidized malaria services. Vouchers covered malaria testing, treatment, and transport to health facilities. The Supportive Supervision Model was developed to enhance the capacity and motivation of CHWs, who play a crucial role in delivering malaria services in hard-to-reach areas.

**Discussion:**

The co-creation process was key to developing contextually relevant and community-owned malaria interventions. It led to three innovations: the CoHPA model, which introduced internal community-led accountability mechanisms; a Health Voucher System that addressed both financial and transport barriers to care; and a supportive supervision model that aimed to improve CHW performance through bi-directional feedback and recognition. While each intervention introduced novel, context-sensitive elements, concerns remain about their scalability, sustainability, and integration into existing health systems without continued support and investment.

**Conclusion:**

The co-creation process produced three community-driven interventions with potential to break key barriers in access to malaria case management in conflict-affected communities of Cameroon. Pilot implementation and community buy-in for integration into national health systems are essential next steps.

## Background

Malaria remains a leading cause of morbidity and mortality in sub-Saharan Africa, with Cameroon among the 11 highest-burden countries [1]. The 2024 WHO World Malaria Report estimated 234 million malaria cases in Africa, with Cameroon’s vulnerable populations especially those in conflict-affected regions bearing a disproportionate share of the disease burden [2]. Since 2016, protracted armed conflict in Cameroon’s North West and South West (NWSW) regions has displaced over 679,000 people, disrupted essential health services, and intensified malaria transmission in overcrowded and under-resourced settlements [3–5]. Amidst this crisis, traditional health system structures have struggled to deliver timely malaria care. Displacement, insecurity, and poverty have compounded access challenges, particularly for women and children [6, 7]. For malaria specifically, these disruptions have led to delays in diagnosis and treatment, increased reliance on informal care, and heightened vulnerability among internally displaced persons living in overcrowded settlements with poor sanitation [6, 7]. In response, we conducted an implementation science research to “Break Barriers” to malaria services. The research project implemented co-created community-based solutions to improve access to malaria services in 80 conflict-affected communities in the South West and Littoral regions of Cameroon [8].

Co-creation has increasingly been recognized in global health as a strategy to develop interventions that are locally appropriate, context-sensitive, and more likely to be sustained [9]. It entails a collaborative process where diverse stakeholders, including communities, jointly design, test, and refine health strategies. Co-creation challenges top-down models by embedding shared ownership, cultural relevance, and mutual accountability into programme development [9–11]. Evidence from global health interventions shows that co-created approaches enhance acceptability and adoption, particularly in settings where trust in the health system is fragile or fractured [9, 12–14].

In humanitarian settings, co-creation takes on additional importance given the complexity of delivering services amid instability, displacement, and insecurity [15]. Humanitarian health interventions often face time pressures, limited resources, and insecurity [16]. Co-creation allows affected communities to articulate their needs, reshape service models, and build trust with implementing partners. Involving communities in design not only fosters legitimacy but also surfaces context-specific innovations that may be overlooked in conventional programming [17–19]. Despite logistical challenges, co-creation has proven to be a viable approach for navigating sensitive political, social, and environmental constraints in crisis-affected contexts.

The conflict in the North West and South West Regions of Cameroon amplified pre-existing barriers to malaria care and created the need for adaptive, community-based co-created solutions. To address barriers to malaria care and treatment, we conducted an implementation science research study aimed at developing, implementing and evaluating scalable, replicable, and innovative approaches to improve access to effective malaria case management through community-based services in conflict affected and host communities in the South-West and Littoral regions of Cameroon. In this manuscript we describe a co-creation process used to collaboratively design and pilot three community-led innovations aimed at breaking barriers to malaria services. This article also explores how participatory design can fosters development of scalable, context-specific solutions that were tested in an implementation science research in conflict-affected communities in Cameroon.

## Methodology

The co-creation process was conducted using an action research approach which involved a single cycle planning, acting and observing, and reflecting [15, 20].

*The planning phase*: the process of co-creating the interventions began with a comprehensive formative research phase conducted in 2021. This formative research included a mixed-methods study combining a Knowledge, Attitudes, and Practices (KAP) survey, malariometric assessment [6], focus group discussions (FGDs), and in-depth interviews [7] across 80 conflict-affected communities in the South West and Littoral regions of Cameroon. These studies have been previously published [6, 7]. The aim of the formative research was to identify local perceptions of malaria, assess barriers to accessing malaria treatment, and explore community trust in existing health services and community health workers (CHWs). Results from the formative study revealed significant challenges including distrust in CHW services, financial and transport barriers, stockouts of essential medicines, and the fragmentation of service provision [6, 7].

*The action and observing phase:* building on the formative research insights, a two-day co-creation workshop was held on the 21 st and 22nd of October 2021 in Buea, South West Region. The workshop aimed to collectively design contextually appropriate intervention strategies that addressed the barriers identified during the formative research. It included plenary sessions to review the findings from the formative research, group work to brainstorm solutions, and consensus-building exercises to refine proposed intervention strategies.

*The reflection phase*: following presentations and brainstorming on potential intervention strategies, participants from the community reflect on their role, share experiences and guidance interventions. Community representatives also voted on intervention naming, and propose implementation modalities. The inclusive and iterative nature of the process ensured that the intervention strategies reflected community priorities, strengthened ownership, and enhanced the likelihood of sustainability in a complex humanitarian setting.

The co-creation workshop was facilitated by two researchers and three humanitarian professionals. The workshop discussions were captured through facilitation notes. Ethics and administrative approvals were obtained prior to conducting our study.

### Participants

The workshop brought together a wide range of stakeholders (41 stakeholders; 24 men and 17 women) from 05 divisions of the South West and Littoral regions (Table 1). Participants were purposefully selected through nominations by local health authorities and community representatives. Participants to the workshop had previously been involved in malaria prevention or treatment services or had been affected by malaria (for host, returnee and IDPs). While regional and district health authorities provided insights to strengthen policy direction and technical approaches. Community health workers and leaders representing host, returnee and IDPs brought perspectives from the community relating to what could work and accepted by the communities. All participants were reimbursed transportation and accommodation fees (for non-resident participants).

**Table 1:** Co-creation workshop participants

Role in community	Men	Women
IDPs	4	3
Hosts	1	1
Returnees	1	1
Community health workers	3	4
Regional health authorities	11	7
Field supervisors	4	1
Total	24	17

### Co-creation process

The process began with a presentation of the entire research project and formative research results. The formative research constituted of a mixed methods study. The quantitative study involved cross sectional survey with 2386 participants and a malariomatric assessment for children under five years (n = 1543) [6]. Meanwhile the qualitative study involved twenty-nine focus group discussions and eleven in-depth interviews [7]. A two-page summary of the results of this research was given to the participants highlighting key findings. This was followed by presentations on three models of community dialogue interventions namely; village health committees, community dialogue approach (CDA) and community development committee. The Community dialogue approach was chosen as the model that could work in the study areas, however with some modifications. Key principles of CDA; transparency and trust, sensitization, collaboration, commitment, active participation, impact and action sharing, community ownership were shared. A key remark arose from the conversation highlighting that the standard dialogue structures put in place by the Ministry of Health had a similar terminology and might be confusing. The different representatives of the Regional Delegates of Public Health, Malaria Regional Technical Group, District Medical Officers and chiefs of health areas provided clarity on the standard dialogue structures and why they were not fit for purpose in the context of the conflict and that of the research (Table 1).

Due to the similarity in name of the innovative community engagement approach which had to be tested in the project and that of the standard dialogue, a different name (community health participatory approach–CoHPA) was adopted by the participants through voting to avoid confusion during project implementation. The main activities to be considered during the implementation of the CoHPA intervention were deliberated upon by participants and the following steps were agreed as main states during implementation (Table 2).

**Table 2:** Differences between the Standard Dialogue structure and the CoHPA

Standard Dialogue Structure	CoHPA
Similarities	
Similarities between standard dialogue and CoHPA	
All discussions centred on promoting primary healthcare within their respective	
Community leaders take the lead in the discussions	
Takes place within the communities	
Held monthly	
Differences	
There is social exclusion	There is social inclusion
Top–bottom approach	Bottom-top approach
Lack of ownership by the community	Owned and managed by the community with opportunities to - adapt topics subject to community concerns and information needs - create discussions that focus on addressing needs of the marginalised and gender difference
No development of action plan after each community dialogue session	Development of action plans by the participants which is evaluated in the next meeting
No evaluation of each meeting and action plans developed	Evaluation of the quality of each meeting by some key participants

During the workshop, District Medical Officers and Chiefs of Health Centres presented the existing supervision mechanisms for Community Health Workers (CHWs), highlighting strengths and gaps. To improve the quality of services delivered by CHWs particularly to strengthen community trust and enhance access to essential malaria treatment, a modified model of “supportive supervision” was proposed, discussed, and formally adopted. Discussions centered on practical implementation modalities, including the designation of responsible personnel for supervision, the frequency of supervisory visits, and the methods of evaluation to ensure accountability and measurable impact. It was also agreed that a baseline assessment would be conducted to test the competencies of CHWs prior to rolling out the new supervision model (Table 3).

**Table 3:** Main activities of CoHPA

Preparatory stage	Implementation stage	Monitory and evaluation
- Co-creation workshop - Identify stakeholders - Meeting with stakeholders - CHW training - Develop materials - Identify participants - Community visit - Sensitization	- Sensitization - Find a comfortable and accessible meeting venue - Community health discussions - Supervision	- Designed scorecard - Pre-testing - Use of scorecard for evaluation

In addition to discussions on supportive supervision, the introduction of health vouchers emerged as a complementary strategy to address financial and logistical barriers to accessing malaria treatment, a key challenge identified during baseline studies (Table 4). Stakeholders acknowledged that despite improved supervision of CHWs, many families will still face challenges in reaching health facilities or affording treatment, especially for children at high risk in displaced and returnee households and or in areas with poor transport links. Through participatory dialogue, three types of vouchers were proposed and adopted which was to be managed entirely at community level by the CHWs:

- *Severe malaria vouchers* targeting children aged 0–5 years to cover the cost of severe malaria treatment. The CHWs had the full responsibility of identifying and referring the patients to the health facilities
- *Transport vouchers* to support the timely referral and transportation of these severe cases to appropriate health facilities after signing memorandum of understanding with them and
- *Simple malaria vouchers* to facilitate early access to care for uncomplicated cases at the community level for all age groups. The decision to adopt these three types was based on practical case scenarios shared during the workshop, data from previous malaria interventions, and an agreement that a tiered voucher system, managed and referred by community health workers would ensure equity and targeted support for those most at risk.

**Table 4:** Difference between standard supervision and supportive supervision

Standard supportive supervision	Modified Supportive supervision
- No baseline conducted to assess competencies of CHWs before supervision tools are developed	- Baseline conducted to ascertain competencies of CHWs before assessment tools developed. CHW performance tracked to identify priority supervision visits and track performance
- No observation of CHW performance as a supervision strategy	- Observations conducted by supervisors and capacities reinforced based on weaknesses observed. Patient audits and attendance at community meetings. Guidelines and refresher training on supportive supervision
- No feedback from CHWs to supervisors	- Feedback is provided by CHWs to supervisors on the supervision process from CHWs and community

### Consensus and validation

Consensus was arrived at during the workshop by voting on key action points to be undertaken by the research team when refining the interventions to be tested in the research. Table 5 presents details they key sessions held during the workshop and action points developed and agreed by participants for consideration in the intervention of the research.

**Table 5:** Summary of activities and action points during discussions

Item	Activities
Group sessions	• Community dialogue approach (CDA) and Community dialogue structure in Cameroon This was a working session in which the members were divided into groups. The following good principles were gathered from them with the help of the facilitator: • Transparency and trust • Sensitization • Collaboration, commitment • Active participation • Impact and action sharing • Ownership Conversely to these good practices, other principles which are detrimental to community-based interventions were equally outlined as bellow: • Dishonesty of actors • Discrimination in selecting beneficiaries
Action point	•To involve different stakeholders at each stage of the designs and programming of activities
Presentation and discussion social behavior change	Presentation on community engagement • What it is? • Why is it important? • Who are the key actors?
Action points/recommandations Action points/recommandations	• Choose leaders that are resident, master common language, of good morals and have good communication skills • Stakeholders involved in CDA and their roles were stated and a point was raised that since the population mostly affected by malaria is pregnant women and under 5 children who are almost always with their mothers, ensuring that a majority of the community key stakeholders are women will ensure greater uptake of services • Barriers to uptake of Malaria services such as poverty was listed among others • Do not reproach the people’s cultures and beliefs but rather understand them to help guide them out of their negative practices and myths • Design interventions that will ensure sustainability • The community members should have a say on the actions to implement • Actors should be chosen among the community members • There should be a harmonized motivation for CHWs • Capacities of CHWs should be strengthened • Financial motivation of CHWs to avoid them selling drugs or acting as community doctors • Meet the right stakeholders • The community should choose the leaders through election • Get the opinion of the community on what they want • Mastery of the subject matter and a suitable venue and time of meeting should be agreed upon intervention to be successful • There should be social inclusion in which, the vulnerable, handicap and social groups are all represented • There is need for harmonization of all CHWs to avoid each implementing organization having their own different CHWs
Resolution	• A new name was selected for this approach, chosen by vote as, Community Health Participatory Approach (CoHPA)
Introduction to community scorecard approach	• What it is? • Why and how is it used?
Action point	• Identify indicators for the use of community scorecards
Scorecard group sessions	Workshop session on the following subject matters were discussed and the results outlined as action points: • Subject and scope • Performance and criteria • Scoring system • Support and needs
Action point (s)	• Service providers: Health personnel, CHWs • Service users: IDPs, other at risk groups and communities • Partners: Administrators, councils, other NGO • Onsite Trainings for facilitators • Tools: Data collection tools, hard copy manuals, stockholder’s matrix, communication tools, didactic material • Sensitization to adopt strategy according to the community • Visual and numeric scorecard matrix will be used to evaluate the interventions • Score monthly at the beginning of the intervention for the first 4 to 6 months, then score quarterly or semesterly
Introduction to supportive supervision	• Exploring the concept of Supportive Supervision to CHWs
Action points	• Improve communication between CHWs and health facilities • Continuous capacity building of CHWs by supervising health facilities • Motivate and encourage CHWs so they have passion for their work by the use of certificate of recognition, badges, standing ovation among other non-financial benefits such a mentoring and resourcing

### Ethics

Ethical approval for the research was obtained from the Institutional Review Board of the Faculty of Health Sciences of the University of Buea (Reference Number: 2021/1286–02/UB/SG/IRB/FHS).

Administrative authorization was obtained from the Southwest Regional Delegation of Public Health and the District Health Services in all study sites. Written and verbal informed consent was sought from all study participants.

### Lessons learned

Three major innovations were co-developed during the workshop: CoHPA was named by participants following consensus that it was better adapted for the conflict context. It was agreed that facilitation of the CoHPA sessions will be done by two community health volunteers (CHVs), a male and a female, selected by the community after sensitization on the qualifications of the CHVs by Reach Out Cameroon. Key criteria were: community members who had lived in the communities for at least 05 consistent years, had at least Advanced Level GCE, good facilitation skills and an interest in health care.

To address financial and physical access barriers, a monthly voucher system was co-created and agreed on during the workshop. This was tailored to community-specific malaria burdens. Vouchers covered malaria diagnostic testing, treatment for uncomplicated and severe cases, and transportation to health facilities.

Recognizing CHWs as frontline actors, a supportive supervision model that blended mentorship, skills reinforcement, non-financial incentives, and retro-feedback from CHWs to their supervisors on how supervision went. Supervisory visits focused on coaching, data quality checks, and technical feedback.

## Discussion

The participatory co-creation process was central to the success of the three co-created innovative interventions. By putting the communities at the centre of problem identification and shifting solutions to them as well, the research enhanced the process of relevance, ownership, and suitability of its interventions for the context.

Following the two-day workshop, three community-based interventions were co-created. The Community Health Participation and Accountability (CoHPA) model was developed to address structural barriers to community engagement in local solutions by enabling local actors to lead dialogues and monitor collective action. Its inclusive structure aimed to challenge entrenched gender norms and promote representation of displaced and vulnerable groups. While similar models of community engagement have been documented in the literature [9, 11, 13, 21, 22], particularly in participatory health governance and social accountability frameworks [14, 23], CoHPA is, to our knowledge, the first to incorporate a mechanism through which community members systematically self-evaluate their performance on agreed malaria-related actions, assess frontline health worker service delivery, and co-develop solutions. This focus on internal accountability, rather than solely upward accountability to external actors, represents a significant shift in how community engagement is operationalised in at risk settings [24]. However, questions remain about the scalability and sustainability of such self-assessment mechanisms without external facilitation or sustained funding.

The health voucher system was co-designed to address financial barriers and promote earlier and more consistent care-seeking. Unlike existing voucher systems which often focus narrowly on reducing user fees at point of care [25–27], this model integrates local realities of transport costs and access constraints, both of which were repeatedly cited by participants as major deterrents to timely treatment. The innovation lies in combining a conditional benefit (subsidised care) with a context-specific barrier (transportation), though the long-term feasibility of subsidising both elements within constrained health budgets remains uncertain.

Finally, the supportive supervision approach was introduced to address critical gaps in community health worker (CHW) performance and retention. By incorporating bi-directional feedback, recognising CHW contributions, and focusing on skill reinforcement rather than fault-finding, the model attempted to reposition supervision as a motivating, rather than punitive, process. While such an approach aligns with global recommendations on CHW support [28–31], its effectiveness will depend on how well it can be institutionalised within existing supervisory structures and whether supervisors themselves are adequately trained and incentivised to adopt a more facilitative role.

## Limitations

The co-creation process had some limitations. First, the purposive selection of participants for the workshop may have introduced selection bias. While efforts were made to ensure diversity by including participants from different community groups, health worker cadres, and local organisations, some voices, particularly from more marginalised or remote communities, may have been underrepresented. This could affect the broader applicability of the co-created interventions, as the outputs may reflect the perspectives of those more accessible or vocal, rather than a comprehensive cross-section of all stakeholders. Nonetheless, the relevance of the co-created models is strengthened by the fact that discussions were grounded in findings from prior formative research and included participants from multiple geographic regions and sectors.

Second, the co-creation process was resource- and time-intensive. Convening stakeholders in person, conducting preparatory qualitative studies, and facilitating multi-day workshops required substantial logistical coordination and financial investment. These demands may limit the replicability of such a process in other humanitarian settings where time-sensitive decisions and resource constraints are common. While alternative methods such as virtual consultations, Delphi panels, or structured interviews could reduce costs, they often lack the interpersonal depth and iterative negotiation necessary for genuine co-production.

## Conclusion

The *Breaking Barriers* co-creation process illustrates the value of collaborative design grounded in community participation to address health service barriers in conflict-affected settings. Through an inclusive and iterative approach, three context-specific interventions; CoHPA, the Health Voucher System, and Supportive Supervision were co-developed to tackle structural, financial, and workforce-related challenges in malaria care. In settings where formal structures are fragile or absent, such participatory processes are not only feasible but essential. As humanitarian crises become more protracted and complex, this experience from Cameroon reinforces the need to design health interventions *with* affected communities, ensuring that solutions are responsive, sustainable, and grounded in lived realities.

## Data Availability

Not applicable.
